# Modeling the Mechanical Consequences of Age-Related Trabecular Bone Loss by XFEM Simulation

**DOI:** 10.1155/2016/3495152

**Published:** 2016-06-15

**Authors:** Ruoxun Fan, He Gong, Xianbin Zhang, Jun Liu, Zhengbin Jia, Dong Zhu

**Affiliations:** ^1^Department of Engineering Mechanics, Jilin University, Nanling Campus, Changchun 130025, China; ^2^Department of Automobile and Construction Engineering, Beihua University, Jilin 132013, China; ^3^Hand & Foot Surgery and Reparative & Reconstructive Surgery Center, No. 2 Hospital of Jilin University, Changchun 130025, China; ^4^Department of Traumatic Orthopedics, The First Hospital of Jilin University, Changchun 130025, China

## Abstract

The elderly are more likely to suffer from fracture because of age-related trabecular bone loss. Different bone loss locations and patterns have different effects on bone mechanical properties. Extended finite element method (XFEM) can simulate fracture process and was suited to investigate the effects of bone loss on trabecular bone. Age-related bone loss is indicated by trabecular thinning and loss and may occur at low-strain locations or other random sites. Accordingly, several ideal normal and aged trabecular bone models were created based on different bone loss locations and patterns; then, fracture processes from crack initiation to complete failure of these models were observed by XFEM; finally, the effects of different locations and patterns on trabecular bone were compared. Results indicated that bone loss occurring at low-strain locations was more detrimental to trabecular bone than that occurring at other random sites; meanwhile, the decrease in bone strength caused by trabecular loss was higher than that caused by trabecular thinning, and the effects of vertical trabecular loss on mechanical properties were more severe than horizontal trabecular loss. This study provided a numerical method to simulate trabecular bone fracture and distinguished different effects of the possible occurrence of bone loss locations and patterns on trabecular bone.

## 1. Introduction

Given rapid increase in the elderly population, age-related fracture has become an important public health issue [1, 2]. Many reasons explain why the elderly are susceptible to fracture; however, the main reason is the decreased bone strength caused by age-related trabecular bone loss [3, 4]. Investigating the effects of trabecular bone loss on the mechanical properties of bone structure may therefore assist in understanding the bone degeneration and fracture mechanism, which is meaningful for preventing age-related osteoporosis and fractures.

Age-related changes in the microstructure of trabecular bone, such as decrease in trabecular thickness and loss of trabecular connectivity, may decrease bone volume fraction (BV/TV) and weaken bone microstructure [3, 5]. Given that trabecular bone contributes significantly to bone mechanical integrity and determines bone quality, the effects of age-related changes in trabecular bone on its mechanical properties have been investigated [6–8]. Trabecular number and thickness decrease with age, and the related bone strength decreases by 34% to 47% among the elderly [7]. In the lumbar spine, rod-like trabeculae become thinner with age and finally disappear, thereby causing a discontinuity in the microstructure [8]. In the femoral head, changes occur in the form of a simultaneous thinning and perforation of the plate-like trabeculae, which lead to new and relatively thinner plates and rods [9]. All these changes constitute the key factors that contribute to the age-related fracture. In addition, trabecular connectivity may also have an important role in maintaining bone strength, but the connectivity inevitably decreases with age, which in turn increases the risk of fracture for the elderly [10].

Since significant relationship between age-related changes in trabecular microstructure and its fracture risk has been found, it is therefore important to understand the effects of such changes on the mechanical properties of trabecular bone [11, 12]. To investigate the effects of various architectural deterioration factors on fracture characteristics, many studies focused on quantifying and comparing the morphological parameters of aged trabecular bones based on microcomputed tomography (micro-CT) images [5, 13]. However, in order to fully understand the effects of age-related changes, it is not sufficient to merely compare the morphological parameters for aged specimens. The degeneration process of trabecular bone with respect to trabeculae type and microstructure should also be investigated. With the development of structure modeling technique, several methods in modeling trabeculae microstructure were put forward. Individual trabecula segmentation technique, which can decompose the trabecular bone network into individual trabecular plates and rods, was developed [14, 15]; meanwhile, a method for subdividing a trabecular network into horizontal and vertical oriented trabeculae was also put forward [8]. Although the type and orientation of trabeculae can be distinguished using above methods, it is also difficult to identify the changes in actual aged trabecular microstructure compared with the normal one. For example, bone loss locations and patterns in actual aged trabecular microstructures may not be determined. Instead, ideal trabecular bone model, which could artificially produce different bone loss locations and patterns for aged trabecular bone models based on the actual degeneration process, can solve this problem [6, 16]. Thus, ideal trabecular bone model can serve as a promising model to investigate the age-related changes in trabecular bone microstructure [17, 18].

In addition, finite element (FE) method has become a highly efficient technique to estimate bone strength and fracture risk [1, 19]. Several FE methods based on fracture analysis could accurately simulate the fracture process, such as the element deletion techniqueand the cohesive zone elements [20, 21]. As a new method of fracture analysis, XFEM can simulate whole fracture process from crack initiation to complete failure more conveniently because its fracture process is independent of mesh and does not require defining a crack-extension path in advance; meanwhile, a quantity of simulative results obtained through XFEM analysis are consistent with the experimental data [19, 22, 23].

Accordingly, this study aimed to simulate the fracture processes of ideal trabecular bone models based on XFEM analysis. Several ideal normal and aged trabecular bone models were first created based on different bone loss locations and patterns, and then the effects of these different locations and patterns of age-related bone loss on the mechanical properties of trabecular bone were compared. These simulations may assist in explaining the age-related fracture mechanism by analyzing the variations in trabecular microstructure and provide a theoretical basis for prevention of age-related fracture.

## 2. Materials and Methods

### 2.1. Construction of Trabecular Bone Models

**Figure 1:**
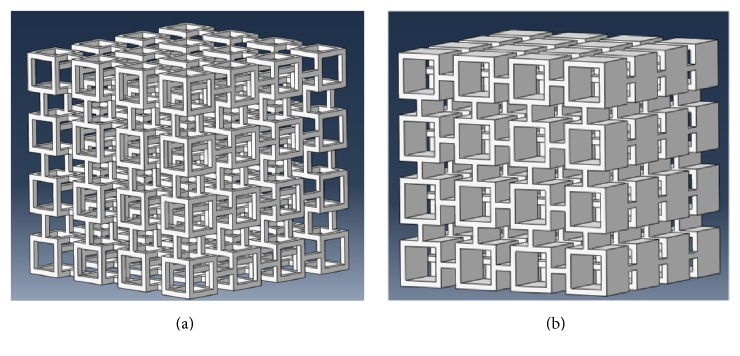
Two ideal normal rod-like and plate-like trabecular bone models were created. (a) Model-rod A with BV/TV of 10.29%. (b) Model-plate A with BV/TV of 27.69%.

Trabecular bone is composed of trabeculae in the form of rods and plates, and trabecular bone microstructure may vary across anatomical locations [9, 24]. Accordingly, two ideal normal trabecular bone models (Model-rod A and Model-plate A) were first created [18, 25–27] (Figure 1). The trabecular bone tissue material was assumed to be isotropic with the elastic modulus of 12 GPa and Poisson's ratio of 0.3 [20, 28].

**Figure 2:**
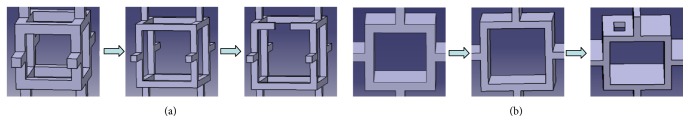
The process of age-related bone loss includes two steps: thinning of the trabecula and its eventual loss. The two-step degeneration process for single rod-like and plate-like trabecular bone cells was shown, respectively. (a) Degeneration process of the rod-like cell: trabecular rods at the two degenerated regions initially became thinner, after which part of these thinner rods were broken. (b) Degeneration process of the plate-like cell: trabecular plates at the two degenerated regions became thinner gradually; then part of these thinner trabecular plates were perforated.

Aging may result in trabecular bone loss at two types of regions: random sites and low-strain locations [6, 16, 29]. Here trabecular bone loss at random sites was defined as degeneration location 1, and trabecular bone loss at low-strain locations was defined as degeneration location 2. Meanwhile, the process of age-related trabecular bone loss is formed in two steps. For rod-like structures, trabecular rods at the two degenerated regions initially became thinner, after which part of these thinner rods were resorbed (Figure 2(a)); given these changes, BV/TV decreased by 15%–20% [9, 30]. For plate-like structures, trabecular plates at the two degenerated regions initially became thinner, after which part of these thinner plates were perforated (Figure 2(b)); given these changes, BV/TV decreased by 25%–30% [10, 31]. Therefore, two aged rod-like and plate-like models in the two degeneration locations were established. First, based on Model-rod A, thickness of the rod-like trabeculae decreased at random sites and at low-strain locations; a number of breakages were then produced in part of the abovementioned thinner rods. As a result, two aged rod-like models (Model-rod B and Model-rod C) were created. For these two aged models, BV/TV decreased by 15% relative to that of Model-rod A. Similarly, based on Model-plate A, plate-like trabeculae became thinner at random sites and at low-strain locations; a number of these thinner trabecular plates then exhibited perforations. As such, two aged plate-like models (Model-plate B and Model-plate C) were created. For these two aged models, BV/TV decreased by 25% relative to that of Model-plate A.

**Figure 3:**
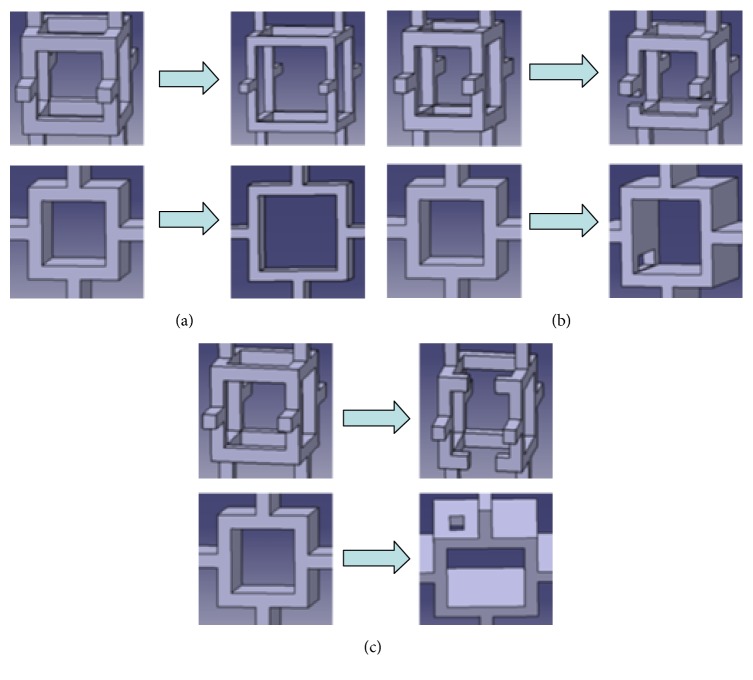
Three degeneration patterns in the ideal trabecular bone models were shown. (a) Degeneration pattern 1: thinning of trabeculae, which were simulated by uniformly reducing the trabecular thickness from the normal models. (b) Degeneration pattern 2: loss of vertical trabeculae, which were simulated by randomly removing the vertical trabecular elements from the normal models. (c) Degeneration pattern 3: loss of horizontal trabeculae, which were simulated by randomly removing the horizontal trabecular elements from the normal models.

Regardless of the locations where trabeculae were lost, the process of age-related bone loss includes two steps: thinning of the trabecula and eventual loss [3, 16, 32]. BV/TV decreases significantly due to trabecular thinning. Trabecular loss has little effect on BV/TV, but it decreases the connectivity of trabecular bone to a great extent. It was unknown whether trabecular thinning would bring severer effects on trabecular bone than trabecular loss in terms of damage and fracture. Thus, it was necessary to compare the relative effects of trabecular thinning and loss on the mechanical properties of trabecular bone. Here trabecular loss was subdivided into loss of trabeculae along the vertical and horizontal directions. Therefore, three degeneration patterns were considered: thinning of trabecula was defined as degeneration pattern 1; loss of vertical trabecula was defined as degeneration pattern 2; loss of horizontal trabecula was defined as degeneration pattern 3. Based on the normal models (Model-rod A and Model-plate A), three sets of rod-like and plate-like models with different degeneration patterns were created: (1) As shown in Figure 3(a), Model-rod D and Model-plate D with degeneration pattern 1 were created by uniformly reducing the thickness of trabeculae from the normal models. (2) As shown in Figure 3(b), a quantity of vertical trabecular elements were randomly removed from the normal models, which formed Model-rod E and Model-plate E with degeneration pattern 2. (3) As shown in Figure 3(c), a quantity of horizontal trabecular elements were randomly removed from the normal models, which formed Model-rod F and Model-plate F with degeneration pattern 3. It was difficult to reduce too much BV/TV through loss of trabeculae alone. Given that the mechanical properties were obviously changed by at least a 5% reduction in BV/TV [16, 33], approximate 8% reduction in the original BV/TV of normal models was simulated for all the different degeneration pattern models.

### 2.2. Fracture Simulation Based on XFEM

The fracture process of trabecular bone structure is generally controlled by strain-based criterion, and both of the tissue yield and crack initiation strains are asymmetric in tension and compression [34–36]. Thus in this paper “Cast Iron Plasticity” model in ABAQUS was used to simulate the asymmetric tensile-compressive tissue yielding. When the tensile or compressive principal strain in the FE model exceeded the tensile or compressive tissue yield strain, the postyield tissue elastic modulus was set to 5% of the initial elastic modulus [37, 38]. In this study, it was assumed that trabecular bone material entered tensile yield stage when its tensile principal strain reached 0.33%, and the tensile crack was initiated when the tensile principal strain exceeded 0.61% and that trabecular bone material entered compressive yield stage when its compressive principal strain reached 0.81%, and the compressive crack was initiated when the compressive principal strain exceeded 1.02% [19, 20, 39].

Although compressive strain may occasionally introduce breakage [40], for XFEM there is no crack initiation criterion in compression in ABAQUS. The subroutine UDMGINI was therefore compiled to embed the compressive crack initiation criterion in this study. Therefore, when either tensile or compressive crack initiation strain in the FE model was exceeded, crack would be initiated, and the new introduced crack was normal to the principal strain direction [23].

Once crack initiation condition was met, crack began to grow obeying bone damage propagation law. The energy-based criterion was selected in damage evolution, and the energy release rates of trabecular bone tissue were all set to 0.33 N/mm [19, 41]. All the models were compressed under displacement-control loading. An apparent strain of 5% was imposed on the top surface of the model, and the bottom surface of the model was constrained. Meanwhile, in this study the average normal and aged trabecular thicknesses were 140 *μ*m and 126 *μ*m [5, 30]. Therefore, considering the mesh sensitivity analysis and solution convergence [38], the average mesh size of 35 *μ*m was selected, and eight-node hexahedron C3D8 element was used.

### 2.3. Validation Experiments

**Figure 4:**
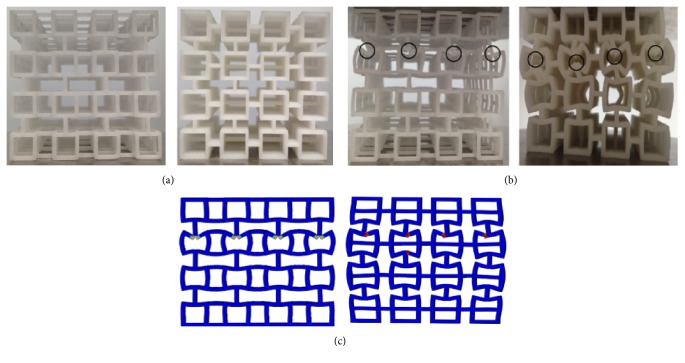
Comparison of the deformations and fracture patterns predicted by the XFEM analysis and those obtained by compressive tests for the corresponding RP models. (a) RP models of Model-rod A and Model-plate A were manufactured by RP laser sinterstation. Each edge of the normal trabecular bone model was scaled up by 10 times in these RP models. (b) The front view of deformations and fracture patterns for the RP models, and the fracture sites were marked by black circle. (c) The front view of fracture contour plots for the FE models.

To validate the method in modeling ideal trabecular bone and XFEM analysis used in this study, four rapid prototype (RP) models of Model-rod A and four RP models of Model-plate A were manufactured, respectively, by RP laser sinterstation (SLS 2500 Plus, DTM Corporation, USA). Due to the limitation of sinterstation resolution, each edge of the ideal normal trabecular bone model was scaled up by 10 times so that trabecular features in the RP models can be observed (Figure 4(a)). Polyamide-12 powder was used as the raw material to manufacture the RP models, and the energy density of the sintering laser was set to 0.016 J/mm^2^ [42, 43]. Then compressive mechanical tests were conducted on these RP models to compare the apparent stress-strain curves and fracture patterns with XFEM simulation for the corresponding Model-rod A and Model-plate A. Here the XFEM simulation was the same with the abovementioned process, and it was assigned with polyamide-12 material instead of trabecular bone material. The elastic modulus of polyamide-12 was set to 1300 MPa, Poisson's ratio was set to 0.3, and yield and crack initiation strains were set to 1.5% and 10%, respectively [44–46].

## 3. Results

### 3.1. Validation of the Ideal FE Models and XFEM Analysis

**Figure 5:**
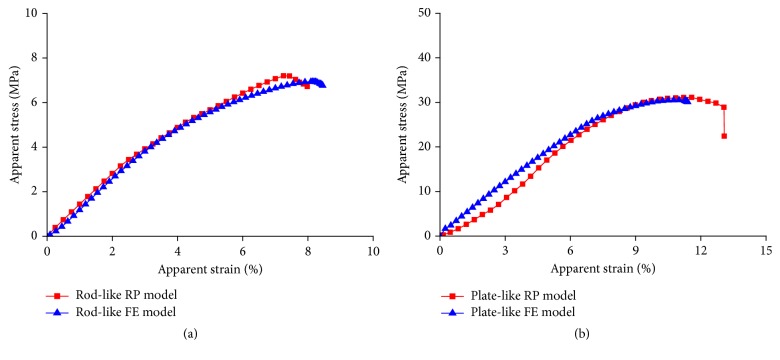
Comparison ofthe apparent stress-strain curves predicted by XFEM analysis for Model-rod A and Model-plate A and those obtained by compressive tests for the corresponding rod-like and plate-like RP models. The experimentally measured curves for the RP models with the same microarchitecture were averaged since no obvious differences in each curve of the same four RP models were observed.

Comparison of the deformations and fracture patterns between the trabecular FE models and the corresponding RP models was shown in Figures 4(b) and 4(c). Because all the RP models were regular, the fracture processes and patterns under compression were nearly the same for the RP models with the same microarchitecture. It can be seen that fractures in both the FE models and RP models with the same microarchitecture appeared at the similar sites, which were at the intersections between vertical and horizontal trabeculae.  Figure 5 shows the comparison of the apparent stress-strain curves predicted by the XFEM analysis for normal FE models and those obtained by the compressive tests for the corresponding RP models. Here the experimentally measured curves for the RP models with the same microarchitecture were averaged since no obvious differences in each curve of the same four RP models were observed. Not only did the predicted stress-strain curves show the same orders of magnitude in fracture strain, that is, the percentage difference between the simulated and experimentally measured fracture strains was less than 8%, but the similar patterns for the curve shapes and onsets of fracture between simulation and experiment were also observed. Thus, these comparisons showed the reliability of the method in modeling ideal trabecular bone and the XFEM analysis used in this study could accurately simulate the experimental results.

### 3.2. Effects of Different Degeneration Locations on Fractures of Rod-Like Models

**Figure 6:**
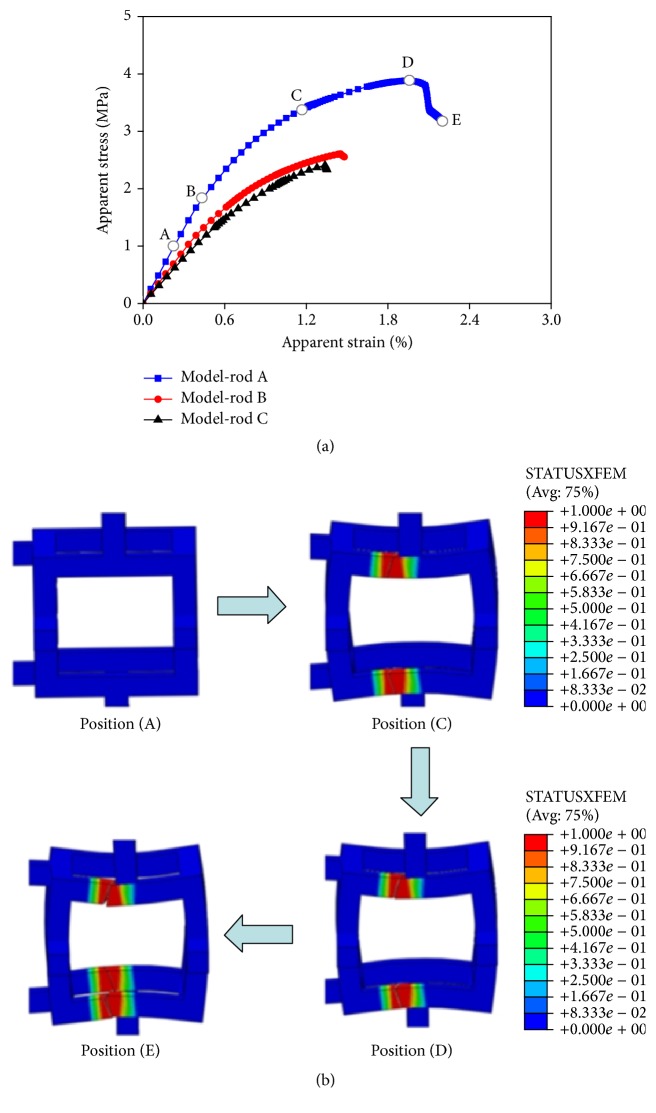
Fracture schematic diagrams of the normal and two aged rod-like models. (a) The apparent stress-strain curves in Model-rods A and B and C. (b) The fracture process from crack initiation to complete failure in Model-rod A.

The apparent stress-strain curves of the three rod-like models were shown in Figure 6(a). Model-rod A had the highest apparent fracture strain and ultimate stress, Model-rod C was the lowest, and Model-rod B was between those two. Regardless of the locations where trabeculae were lost, bone loss resulted in negative effects on the mechanical properties of trabecular bone, particularly for Model-rod C, whose apparent fracture strain and ultimate stress were 30.93% and 37.89% lower than those in Model-rod A. A typical fracture process from crack initiation to complete failure in Model-rod A was shown in Figures 6(a) and 6(b). The deformation started with a linear elastic stage (A) and then entered yield stage (B). The crack was initiated at a quantity of trabeculae (C) and then continued to grow in the stiffness degradation stage, leading to a nonlinear relationship between stress and strain (CD). The stress continued to grow until it reached the ultimate stress (D), which led to a softening stage (DE) for the damaged trabeculae and in turn resulted in a shift from extensively continuous damage to localized permanent failure for the damaged trabeculae. The load was finally carried by the neighboring normal trabeculae, instead of the trabeculae that experienced localized permanent failure, until the complete fracture of the trabecular bone structure (E).

### 3.3. Effects of Different Degeneration Locations on Fractures of Plate-Like Models

**Figure 7:**
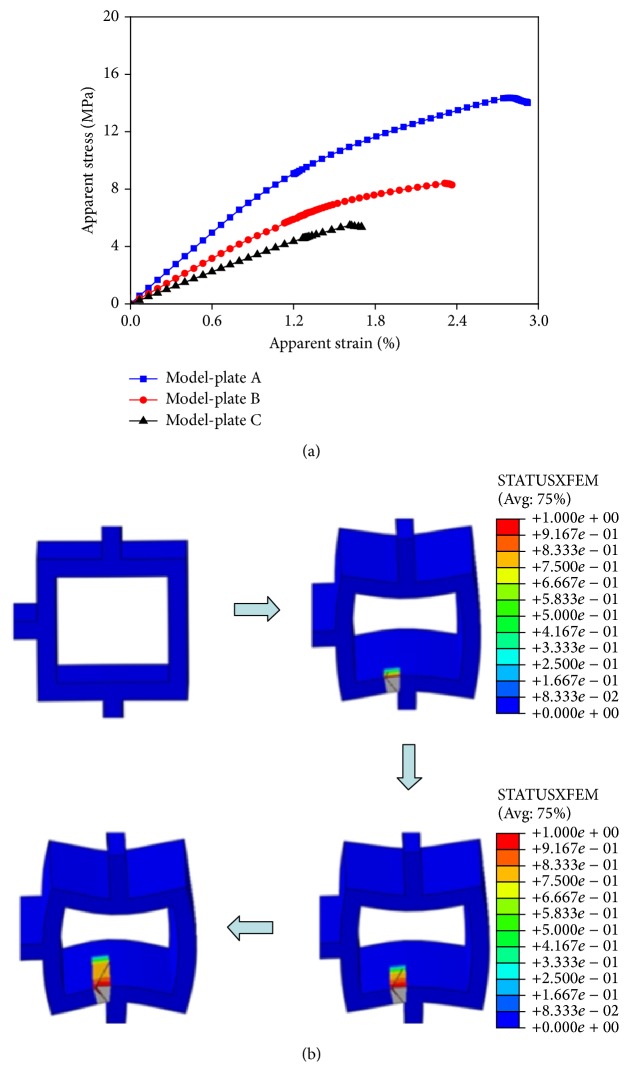
Fracture schematic diagrams of the normal and two aged plate-like models. (a) The apparent stress-strain curves in Model-plates A and B and C. (b) The fracture process from crack initiation to complete failure in Model-plate A.

The apparent stress-strain curves of the three plate-like models were shown in Figure 7(a). The highest apparent fracture strain was observed in Model-plate A (i.e., 2.79%) and the fracture strains of Model-plates B and C were 2.31% and 1.61%, respectively. The apparent ultimate stress also decreased significantly with deteriorated structures, decreasing from 14.36 MPa in Model-plate A to 8.41 MPa and 5.48 MPa in Model-plates B and C, respectively. The fracture process from crack initiation to complete failure in Model-plate A was shown in Figure 7(b). When crack initiation condition was met, a small crack was generated in the plate-like trabecula. With increasing strain, the crack started to grow inside the trabecula along horizontal direction, until complete fracture of the trabecular bone structure occurred.

### 3.4. Effects of Different Degeneration Patterns on Fracture

**Figure 8:**
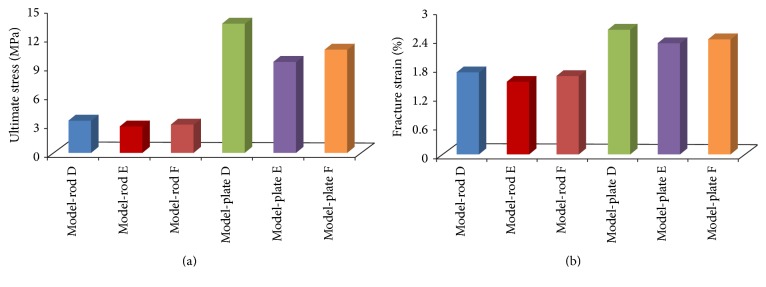
Apparent ultimate stresses and fracture strains of rod-like models and plate-like models in the three degeneration patterns.

**Figure 9:**
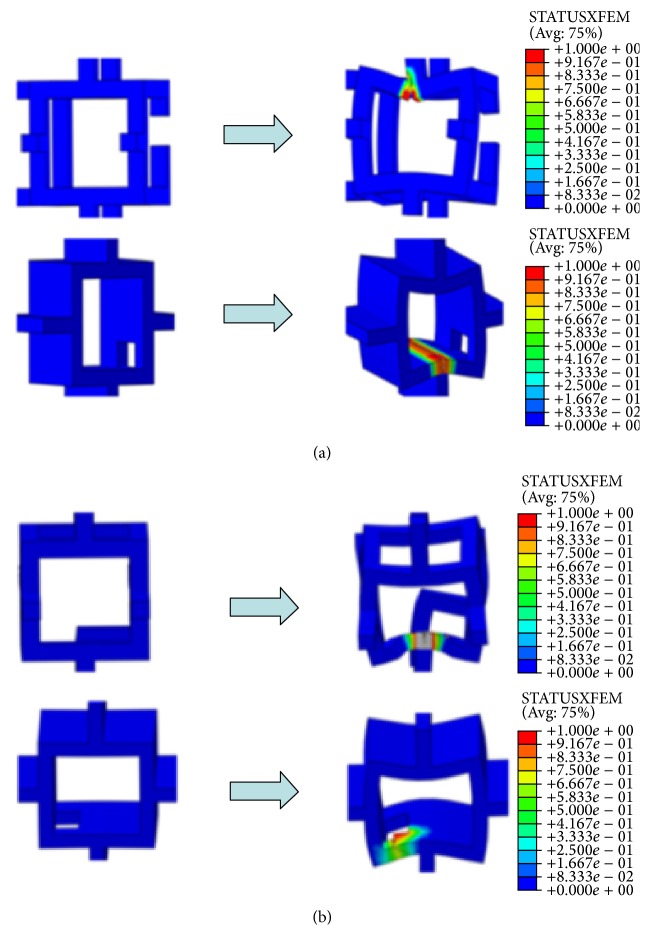
Localized fracture contour plots of trabecular loss models. (a) Typical localized fractures presented in Model-rod E and Model-plate E. (b) Typical localized fractures presented in Model-rod F and Model-plate F.

Different trabecular bone degeneration patterns caused by trabecular thinning or loss both led to decrease in the mechanical properties of normal trabecular bone models, whereas the mechanical properties were more sensitive to trabecular loss than to trabecular thinning; furthermore, the mechanical properties of trabecular bone were less affected by loss of horizontal trabeculae than by loss of vertical trabeculae (Figure 8). For the trabecular loss models, the localized fracture sites were all close to the disconnected trabeculae (Figure 9).

## 4. Discussion

This study utilized a numerical simulation method to predict the fracture processes in normal and aged trabecular bone models. The fractures of these models were simulated using XFEM analysis embedded subroutine UDMGINI, in which the principal strains in tension and compression were used to control the crack initiation and propagation. In the simulation process, when the principal strain in the aged trabecular bone model exceeded the crack initiation strain of trabecular bone tissue, crack in the aged model would be initiated, and then the crack began to grow obeying the bone damage propagation law until complete fracture occurred in the aged model. According to the XFEM analyses in this study, different fracture processes from crack initiation to complete failure for the aged trabecular bone models were primarily observed; then, apparent ultimate stress and fracture strain of the aged trabecular bone models were obtained, and the mechanical properties of different aged trabecular bone models were compared; finally, the effects of bone degeneration locations and patterns on the mechanical properties of aged trabecular bone models were analyzed quantitatively.

At present, many numerical methods can simulate crack initiation and propagation of bone structure [21, 47, 48]. Compared with these methods, XFEM can obtain detailed fracture information at localized damage sites. Because apparent fracture begins with localized damage, investigating the mechanical characteristics at localized damage sites can assist in exploring the mechanism of fracture [13]. For other methods, however, analyzing localized damage of trabeculae at tissue level remains difficult because of the limitation of calculation principle.

In this study, the apparent fracture strain of Model-rod A was 1.94%, and that of Model-plate A was 2.79%. These were consistent with the previous investigation, which shows that the apparent fracture strains are 2% and 2.7% in the normal rod-like and plate-like trabecular bone models [20]. Several experimental results show that the apparent ultimate stress is 3.18 MPa in human lumbar spinal trabecular bone and 3.64 MPa in proximal tibia trabecular bone, which were consistent with our computational results [49, 50]. Meanwhile, the aged rod-like models (Model-rods B and C) were used to represent osteoporotic trabecular bones in this study, and their apparent fracture strains were 1.45% and 1.34%, which were in agreement with the results of compressive experiments for the human osteoporotic trabecular bones [51]. All these comparisons showed the accuracy of the predicted results based on XFEM analysis in this study.

With respect to the roles of rod-like and plate-like trabeculae in fracture process, rod-like models may participate in initiation and progress of fracture at the sites with low bone density, which are more susceptible to large deformation or buckling failure; by contrast, plate-like models may be located at sites with high bone density, which are most likely to bear bending loads [27, 33, 52, 53]. Crack initiation and propagation are correlated with microstructure and BV/TV, and structure model index (SMI) may also be one of the key predictors [54, 55]. Increasing SMI definitely causes negative effects on the mechanical properties of trabecular bone [56]. This conclusion was also supported by our results: the fracture strain and ultimate stress in the plate-like models were all higher than those in the rod-like models. Unfortunately, part of trabeculae are inevitably converted from plate-like to rod-like forms with age, thereby increasing SMI [3, 10]. Such change may be one of the reasons why the elderly are more likely to suffer from fracture.

As shown in Figures 6(a) and 7(a), regardless of the trabecular structures, the lengths of yield stage in the stress-strain curves for the aged models were all shorter than those for the corresponding normal models. Moreover, as the degeneration aggravated, ductile fracture slowly turned to brittle fracture in aged models. This phenomenon was supported in literature: advanced glycation end products, which have been reported to alter the formation and propagation of damage by making the bone more brittle, are produced in human bone with age [57]. Thus, final fracture strains decreased in aged trabecular bone models because of shorter yield stage, resulting in earlier fracture. In addition, it has been reported that trabeculae at low-strain locations (degeneration location 2) are more likely to be resorbed with age [16]. Combining with our observation, the effects of degeneration location 2 on trabecular bone were evidently more severe than those of degeneration location 1. Therefore, compared with bone loss occurring at the other degeneration locations, bone loss occurred at degeneration location 2, which is more likely to be resorbed, and had more serious effects on the mechanical properties of trabecular bone. This phenomenon explained why the elderly were more likely to suffer from fracture from bone loss mechanism.

Regardless of the locations where trabeculae are lost, age-related bone loss is derived from trabecular thinning or loss. Thus, it is necessary to determine the crucial degeneration factor by identifying the relative effects of trabecular thinning and loss on mechanical properties. As shown in Figure 8, loss of vertical trabeculae generated the most serious effects, which was in agreement with the experimental results [58, 59]. Thus, the effects of loss of vertical trabeculae on trabecular bone were tremendous, particularly for rod-like trabecular bone [32]. Given that the load is parallel to the vertically oriented trabeculae, vertical trabeculae will bear most of the load and horizontal trabeculae may act as stabilizing cross-braces, and the vertical trabeculae were therefore more highly strained than the horizontal trabeculae [8, 58]. If the vertical trabeculae are damaged or resorbed massively, the load carried by the intact vertical trabeculae in the vicinity of the damaged vertical trabeculae will increase and accumulate rapidly, which may lead to two consequences. First, if the intact vertical trabeculae in the vicinity of the damaged ones possess large slenderness, the intact vertical trabeculae may result in buckling failure. Buckling failure is recognized as relevant to the failure of the vertebral trabecular bone [33, 42]. Second, if the condition of buckling failure is not met, brittle fracture may occur under relatively small strain for the supporting horizontal trabeculae between two intact vertical trabeculae. Because vertical trabeculae are lost massively, the loads supported by the remaining vertical trabeculae are too large, which may produce too much bending on the cross-bracing horizontal trabecula between them. Both of these phenomena are consistent with the conclusions that failures in the vertical trabeculae are predominantly generated by compressive deformation, whereas failures in the horizontal trabeculae are predominantly generated by bending [16, 59]. Thus, these two consequences may be highly correlated with the fracture of rod-like trabecular bone structure.

For the plate-like structures, trabecular loss was generated by resorption cavities, and the connectivity within the structure was not totally lost, so that the load can still be carried and transmitted, which had better mechanical properties compared with rod-like structures [15]. However, this type of resorption was characterized by a chain effect, whereby stress concentrations in the vicinity of the resorption cavities still caused further damage. Therefore, fracture occurring in the aged plate-like trabecular bone models was also caused by relatively small loading. These phenomena suggested the importance of trabecular connectivity in maintaining the mechanical properties of trabecular bone, particularly for the integrity of the vertical trabeculae in rod-like structure.

Several limitations were associated with the ideal trabecular bone models and simulation process in this study. First, there are some differences in microstructure between ideal and actual trabecular bone models. Understanding the effects of different degeneration locations and patterns on mechanical properties of trabecular bone was of great significance. However, actual trabecular microstructure is very complex, which may lead to difficulties in identifying different degeneration locations or patterns in actual trabecular bone models. To overcome this problem, ideal trabecular bone models were used in this study. These ideal models allow artificially inducing bone losses which were manufactured from normal models and direct comparison between the normal and aged models without confounding variations in tissue properties that are inherent in actual trabecular bones [42, 43]. Although the trabecular bone models were idealized, all the age-related changes in the microstructure were developed based on actual degeneration processes, and all BV/TVs were selected according to literature [3, 5, 9, 10, 27, 30]; meanwhile, validation process also showed the reliability of the method in modeling ideal trabecular bone (Figures 4 and 5). Thus, these two types of models can characterize the essence of the microstructural features of actual trabecular bones. Second, isotropic trabecular bone material was used because the aim of this study was to investigate the effects of architectural deterioration on the mechanical properties of trabecular bone, and modeling trabecular bone tissue as isotropic material may not generate a strong effect on the predicted results compared with applying anisotropic material parameters [20, 60]. Finally, the mechanical properties of materials that exhibit stiffness degradation and softening behavior often lead to severe convergence difficulty [20, 22]. Although ABAQUS software provides several viscous parameters to improve the convergence, the convergence problem appeared after complete fracture in certain aged models occurred. However, it had little effects on the results of apparent ultimate stresses and fracture strains.

## 5. Conclusions

Age-related trabecular bone loss occurring at low-strain locations was more detrimental to trabecular bone structure than that occurring at other random sites; meanwhile, the decrease in trabecular bone strength caused by trabecular loss was higher than that caused by trabecular thinning, and the effects of vertical trabecular loss on the mechanical properties of trabecular bone were severer than horizontal trabecular loss.
